# Rotavirus Infection Induces Transient Pancreatic Involution and Hyperglycemia in Weanling Mice

**DOI:** 10.1371/journal.pone.0106560

**Published:** 2014-09-02

**Authors:** Margo C. Honeyman, David Laine, Yifan Zhan, Sarah Londrigan, Carl Kirkwood, Leonard C. Harrison

**Affiliations:** 1 The Walter and Eliza Hall Institute of Medical Research, Parkville, Victoria, Australia; 2 Department of Medical Biology, University of Melbourne, Parkville, Victoria, Australia; 3 Murdoch Children's Research Institute, Royal Children's Hospital, Parkville, Victoria, Australia; La Jolla Institute for Allergy and Immunology, United States of America

## Abstract

Rotavirus is a ubiquitous double-stranded RNA virus responsible for most cases of infantile gastroenteritis. It infects pancreatic islets *in vitro* and is implicated as a trigger of autoimmune destruction of islet beta cells leading to type 1 diabetes, but pancreatic pathology secondary to rotavirus infection *in vivo* has not been documented. To address this issue, we inoculated 3 week-old C57Bl/6 mice at weaning with rhesus rotavirus, which is closely related to human rotaviruses and known to infect mouse islets *in vitro*. Virus was quantified in tissues by culture-isolation and enzyme-linked immunosorbent assay. A requirement for viral double stranded RNA was investigated in toll-like receptor 3 (TLR3)-deficient mice. Cell proliferation and apoptosis, and insulin expression, were analyzed by immunohistochemistry. Following rotavirus inoculation by gavage, two phases of mild, transient hyperglycemia were observed beginning after 2 and 8 days. In the first phase, widespread apoptosis of pancreatic cells was associated with a decrease in pancreas mass and insulin production, without detectable virus in the pancreas. These effects were mimicked by injection of the double-stranded RNA mimic, polyinosinic-polycytidylic acid, and were TLR3-dependent. By the second phase, the pancreas had regenerated but islets were smaller than normal and viral antigen was then detected in the pancreas for several days. These findings directly demonstrate pathogenic effects of rotavirus infection on the pancreas in vivo, mediated initially by the interaction of rotavirus double-stranded RNA with TLR3.

## Introduction

Viruses, in particular enteroviruses, have been implicated as triggers of the autoimmune process that leads to destruction of insulin-producing pancreatic islet beta cells in type 1 diabetes [1], [2]. The first direct demonstration of a link between virus infection and type 1 diabetes was by Yoon and colleagues [3], who showed that beta cell damage and insulin-dependent hyperglycemia followed infection of susceptible mice with Coxsackie virus B4. However, despite intense investigations, including *in situ* evidence for enterovirus infection of human islets [4], a causal relationship remains elusive. Rotavirus (RV) is a ubiquitous double-stranded RNA virus responsible for most cases of infantile gastroenteritis. In a longitudinal study of Australian infants who went on to develop type 1 diabetes RV infection was observed concomitantly with an increase in the titer of autoantibodies to the pancreatic islet autoantigens glutamic acid decarboxylase 65 (GAD65) and tyrosine phosphatase-like insulinoma antigen 2 (IA2) [5], although this association was not confirmed in a subsequent Finnish study [6]. In addition, peptides in the serologically-dominant capsid protein VP7 of RV were found to have strong sequence similarity [7] and cross-reactivity [8] with T-cell epitopes in GAD65 and IA2, implicating molecular mimicry as a mechanism linking RV infection with islet autoimmunity. Nevertheless, the pathogenic significance of molecular mimicry remains unclear and RV-mediated tissue damage would seem a more plausible mechanism initiating autoimmunity.

RV is a double-stranded RNA virus and therefore it is of interest that beta cells express the innate immune toll-like receptor 3 (TLR3) for double-stranded RNA [9], [10], as well as the double-stranded RNA sensor, IFN-induced helicase 1, which is a susceptibility gene for type 1 diabetes [11]. Double-stranded RNA has been reported to induce apoptosis in rodent beta cells [12] and we have shown that several RV strains infect mouse and monkey islets *in vitro* leading to beta cell death [13]. Inoculation of autoimmune diabetes-prone non-obese diabetic (NOD) mice with one of these strains, rhesus RV (RRV), after the onset of islet autoimmunity, accelerated the development of diabetes, but effects on the pancreas or glycemia were not reported [14]. Our aim was to determine if RV infection *in vivo* compromised beta cell function in non-diabetes prone mice. We demonstrate that RRV triggers pancreatic apoptosis and transient involution associated with mild hyperglycemia in C57Bl/6 mice at weaning, the time when humans are most vulnerable to RV infection.

## Methods

### Ethics statement

Experiments were approved by the Animal Ethics Committee of the Walter and Eliza Hall Institute of Medical Research, Melbourne, Australia.

### Virus

RRV was used because we showed previously that it infected mouse pancreatic islet cells in culture [13]. It is also culture-adapted and therefore can be administered in standardized doses. RRV was generated in MA104 African green monkey kidney epithelial cells [15], grown to confluence in Dulbecco's modified Eagle medium (DMEM) supplemented with 10% fetal calf serum (FCS), 0.15% NaH_2_CO_3_, 0.03% L-glutamine, 25 U penicillin and 250 µg streptomycin/ml, at 37°C in 5% CO_2_ 95% air, and transferred to serum-free medium 24 h prior to infection. The virus was activated by incubation with porcine trypsin (10 µg/ml; Sigma-Aldrich, Sydney, Australia) at 37°C for 30 min and MA104 monolayers were infected by adsorption of the activated RRV for 1 h at 37°C. The inoculum was then removed and infected cells were incubated in serum-free DMEM containing 1 µg/ml trypsin until a complete cytopathic effect was evident (within 24 h). Cultures were freeze-thawed three times and cell debris removed by centrifugation at 5000 rpm for 10 min at 4°C. Virus titers expressed as fluorescence focus forming units/ml (FFU/ml) were determined in MA104 cells as described [15]. After incubation with 1/100 biotin-conjugated goat anti-RV antibody (Virostat, Portland, ME) for 1 h at 37°C, followed by 1/500 streptavidin-Alexa 594 (Molecular Probes, Eugene, OR) for 1 h at 37°C, infectious particles were detected by fluorescence in a Zeiss Axiovert 200 M microscope.

Mock preparations were obtained after inoculation of MA104 cells with serum-free DME containing 1 µg/ml of trypsin for an equivalent time. RRV was inactivated by treatment with psoralen and exposure to UV light for 45 min, as previously described [16].

### Mice and inoculation

To mimic human infection, mice were inoculated with RRV or mock virus by gavage, at 3 weeks of age immediately after weaning. To examine innate immune mechanisms in RV infection, TLR3 gene targeted mice on the C57/B6 background [17] were mock or RRV inoculated. Mice were inoculated under methoxyfluorane anesthesia by gavage with 50 µl sodium bicarbonate buffer to neutralize gastric acid, followed 15 min later by 50 µl of RRV containing 10^6^ plaque-forming units (PFU) diluted in TSC buffer (50 mM Tris, 150 mM NaCl, 5 mM CaCl_2_). Mock-inoculated mice received equivalent material from uninfected mouse pellets or MA104 cell lysate. Apart from mild transient diarrhoea in a minority, inoculated mice ate normally and did not appear unwell.

### Treatment with polyinosinic-polycytidylic acid (poly I∶C)

Mice were injected intraperitoneally (i.p.) with 7.5 µg poly I∶C/gm of body weight on day 0. At days 2 and 10 pancreata were harvested, weighed then frozen in Tissue-Tek embedding compound (Sakura, Torrance, CA).

### Blood glucose monitoring and plasma insulin

Glucose was measured at 1300–1400 h after fasting for 6 h with an Accu-Check Advantage meter (Roche Diagnostics, Sydney, Australia) in tail vein blood at different times after inoculation or in blood obtained via cardiac puncture at the time of sacrifice. Mice were considered diabetic if blood glucose was confirmed as ≥ 12.2 mM (mean + 3SD of 280 samples from C57/B6 mice over days 1–14 after mock inoculation). Plasma insulin was measured by enzyme-linked immunosorbent assay (ELISA) (Mercodia, Uppsala, Sweden) in the same tail vein blood sample.

### Measurement of RV by ELISA and titration

At time points indicated, 3–6 mice from RRV and mock control inoculated mice were killed by CO_2_ asphyxiation. Samples were collected in the following order: cardiac blood, pancreas, spleen, liver, mesenteric lymph node and, finally, jejunum (to prevent contamination of peripheral tissues with gut contents). Weighed organs were stored at −20°C in either PBS-5mM CaCl_2_ buffer for ELISA as previously described [18] or in 1 ml ice-cold gelatin-saline (1.7% gelatin, 0.8% NaCl, 0.2 mM CaCl_2_, 0.8 mM MgCl_2_, 20 mM boric acid, 0.13 mM sodium borate) for titration. Infectious RRV particles were isolated from organs as described (19) and virus titers determined on MA104 cells as described above.

### Immunohistochemistry

Organs were fixed with Bouin's solution or 4% paraformaldehyde (PFA) for 24 h at room temperature, then paraffin embedded and serially sectioned. Bouin's fixed sections of pancreas (every 100 µm) were stained with hematoxylin and eosin (H&E), Gomori-aldehyde-fuchsin (GAF, for insulin-zinc complexes in beta-cell granules) and antibodies to insulin (1/200 guinea pig anti-swine insulin, DAKO, Sydney, Australia) or glucagon (1/200 rabbit anti-human glucagon, DAKO) followed by either 1/400 FITC-labeled goat anti-guinea pig antibody (ICN, Sydney, Australia) or 1/400 Alexa 594-labeled goat anti-rabbit antibody (Invitrogen, San Francisco, CA). Incubations with primary and secondary antibodies were for 1 h at room temperature. Pancreas-duodenal homeobox 1 (PDX-1) was detected in PFA-fixed sections incubated overnight at 4°C with 1/20 goat anti-PDX-1 antibody (Santa Cruz Biotechnology, Santa Cruz, CA) followed by 1/200 fluorescein isothiocyanate (FITC)-conjugated rabbit anti-goat immunoglobulin (Dako) for 1 h at room temperature. Proliferating cell nuclear antigen (PCNA) was detected with 1/20 biotinylated mouse anti-human PCNA (Zymed/Invitrogen, San Francisco, CA) for 1 h, followed by 1/400 streptavidin-Alexa 594 (Molecular Probes, Eugene, OR) for 1.5 h at room temperature. Immunofluorescent images were captured with an Axiocam camera from a Zeiss Axioplan2 compound microscope. Terminal deoxynucleotidyl transferase dUTP nick end labeling (TUNEL) staining for apoptotic cells was performed with an Apoptag FITC kit (Chemicon). Staining for CD45 was performed on PFA-fixed sections with rat anti-mouse CD45 monoclonal antibody, clone 30-F11 (Becton-Dickenson, San Jose, CA). The number of islets and TUNEL-positive cells was scored in 10 sections from each mouse, being careful not to recount the same islets or cells.

### Statistical analysis

Results were expressed as mean ± standard deviation (SD) and differences between groups were compared by unpaired t tests (2-tailed). Data were analyzed by PRISM version 6 for Macintosh (GraphPad Software, San Diego, CA).

## Results

### Biphasic hyperglycemia follows inoculation of C57BL/6 mice with RRV

**Figure 1:**
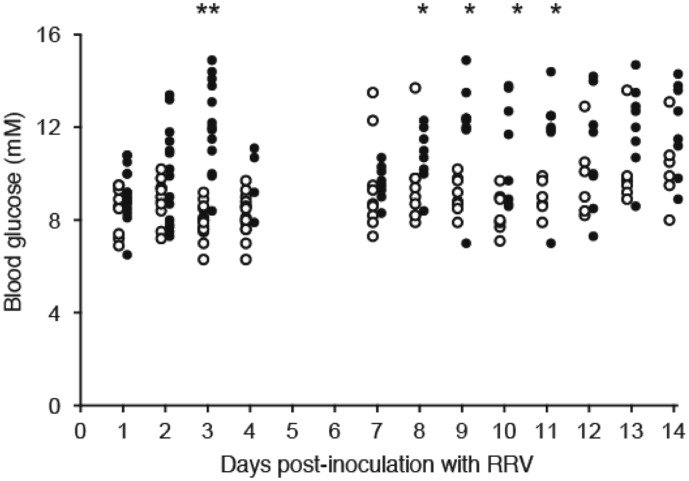
Blood glucose concentrations following RRV or mock inoculation. Blood glucose was measured in individual C57BL/6 mice over 2 weeks following inoculation by gavage with 10^6^ FFU RRV (black circles; 6 females, 6 males) or mock virus (white circles; 4 females, 5 males). * P≤0.02; ** P<0.001. Results are representative of 3 separate experiments.

Following inoculation of wild-type C57/B6 mice with RRV at weaning, blood glucose increased from a mean ± of 8.4±0.96 to 12.6±2.4 mM (P = 0.0001) over 3 days, decreased, and then increased again in a second phase from days 7–11 to a maximum of 12.1±2.3 mM (P = 0.004) at 9 days (Figure 1). Differences in blood glucose between RRV and mock controls were significant at day 3 and days 8–11 (Figure 1). Blood glucose in C57/B6 mice inoculated with mock RRV was occasionally increased in the second phase, but never for more than 24 h (mean 8.8±1.6 mM at days 1–3 and 9.1±1.5 mM at days 7–14). Responses were similar in males and females.

### Acute, TLR3-dependent involution of the pancreas follows inoculation with RRV

**Figure 2:**
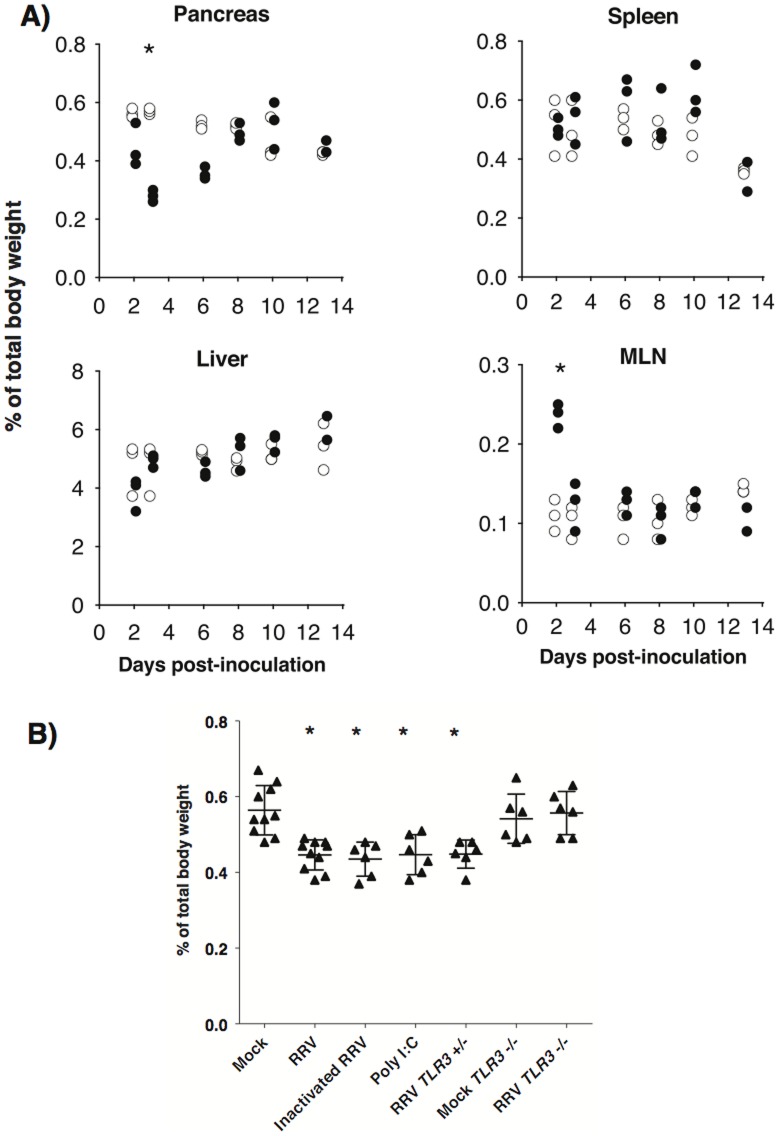
Acute, TLR3-dependent involution of the pancreas following inoculation with RRV. A) Organ weights (% of total body weight) of C57BL/6 mice 3 days after inoculation with RRV (black circles) or mock virus (white circles) (n = 6 mice/group). B) Pancreas weights 2 days after inoculation of C57BL/6 mice with mock virus, RRV or inactivated RRV, or after i.p. injection of poly I:C (n = 6–10 mice/group), or after inoculation of TLR3 gene-targeted mice (n = 6 mice/group). Results in * denotes significant difference from mock virus controls (see text for P values).

By 3 days after inoculation with RRV the pancreas in C57/B6 mice was decreased in size to the naked eye. The pancreas mass (% total body weight) was half that of control mice inoculated with mock virus (0.28±0.02 vs 0.57±0.01, P<0.0001) (Figure 2A). Recovery was rapid and by day 8 the pancreas mass was similar to that of control mice. This effect of RRV was specific to pancreas and was not observed in liver or spleen (Figure 2A). Consistent with enteric infection by RRV, the mediastinal lymph nodes transiently doubled in weight after inoculation compared to mock virus controls (0.24±0.01 vs 0.11±0.02, P = 0.001) (Figure 2A). Live virus was not required to cause involution of the pancreas, which was also observed 2 days after mice were inoculated with psoralen/UV light-inactivated RRV or injected i.p. with the double-stranded RNA mimic, poly I∶C (Figure 2B). Thus, the pancreas mass (% of body weight) after RRV, inactivated RRV and poly I∶C respectively was 0.45±0.04, 0.44±0.05 and 0.45±0.05, compared to 0.56±0.06 after mock virus (P = 0.008).

Double stranded RNA of RV is a natural ligand for TLR3 [19], [20]. To demonstrate a requirement of TLR3 for the effect of RRV on the pancreas, we inoculated *TLR3 +/−* and *TLR3 −/−* mice (on a C57/B6 background) at weaning. Two days after RRV the pancreas mass had decreased in heterozygous *TLR3* +/− mice (0.45±0.04, P = 0.009) but had not changed in homozygous *TLR3* −/− mice (0.56±0.06, P = 1.00) compared to mock virus in *TLR3* −/− mice (0.54±0.06) (Figure 2B).

**Figure 3:**
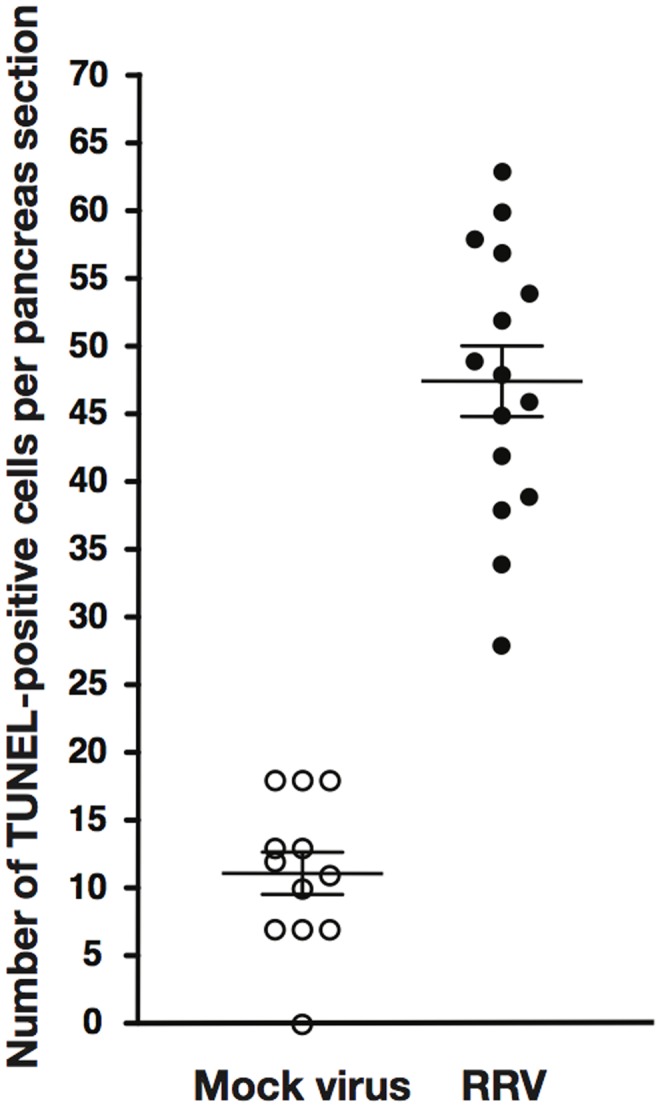
Increase in the number of TUNEL-positive cells in the pancreas following inoculation with RRV. Pancreas sections from C57/B6 mice 2 days after inoculation with mock virus (open circles) or RRV (solid circles) were scored for TUNEL-positive cells per section in 10 sections/mouse. Each point is the total number of TUNEL-positive cells per mouse; the mean and SD are superimposed.

Two days after inoculation with RRV widespread apoptosis detected as DNA strand breakage by TUNEL was present in both the pancreatic exocrine tissue and islets, with no obvious difference in the distribution density. TUNEL-positive cells after RRV were 47.5±10.1 per pancreas section compared to 11.2±5.4 after mock virus (P<0.0001) (Figure 3). Hematoxylin and eosin (H&E) staining of the pancreas on days 2 and 3 revealed no evidence of cellular infiltration (data not shown).

### Islet size and insulin content decrease following inoculation with RRV

**Figure 4:**
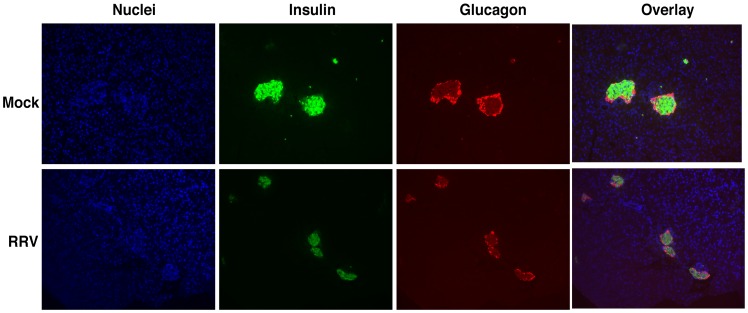
Decrease in islet size and insulin content following inoculation with RRV. Pancreas sections from C57/B6 mice 2 days after inoculation with mock virus or RRV were fixed with 4% PFA and stained with DAPI for nuclei (blue) and with antibodies to insulin (green) and glucagon (red). Magnification x10.

Two days after inoculation with RRV the islet number per pancreas section was similar to mock virus control mice (26.7±11.8 vs 27.0±11.5) but the majority of the islets were less than 50% of the size of islets in the mock control mice, were irregular, and their staining intensity for insulin was decreased (Figure 4).

**Figure 5:**
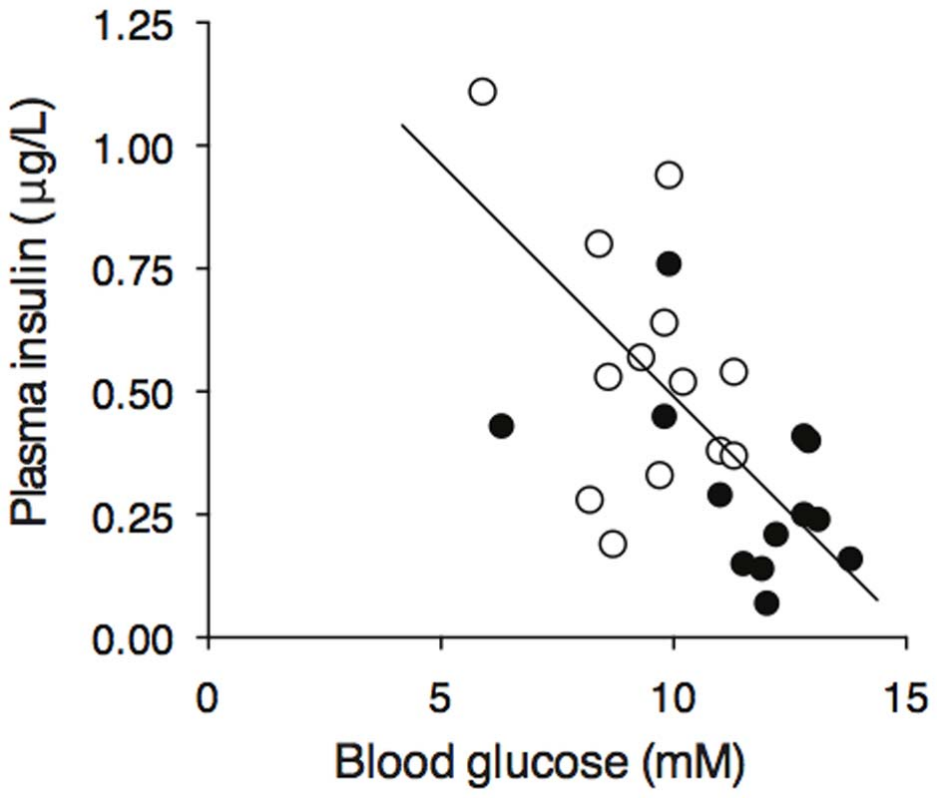
Inverse relationship between plasma insulin and blood glucose concentrations following inoculation with RRV. Plasma insulin and blood glucose were measured in cardiac puncture blood 3 days after inoculation of C57/B6 mice with mock virus (open circles) or RRV (solid circles).

One day after inoculation with RRV plasma insulin concentration was similar to that in mock virus control mice (0.58±0.21 vs 0.56±0.27 µg/l), but by day 3 the increase in blood glucose in RRV mice (Figure 1) was associated with a decrease in plasma insulin compared to the controls (0.33±0.25 vs 0.55±0.26 µg/l, P = 0.016), and plasma insulin and blood glucose concentrations were inversely related (r = 0.56, P = 0.003) (Figure 5).

### Regeneration follows acute RRV-induced pancreas injury

**Figure 6:**
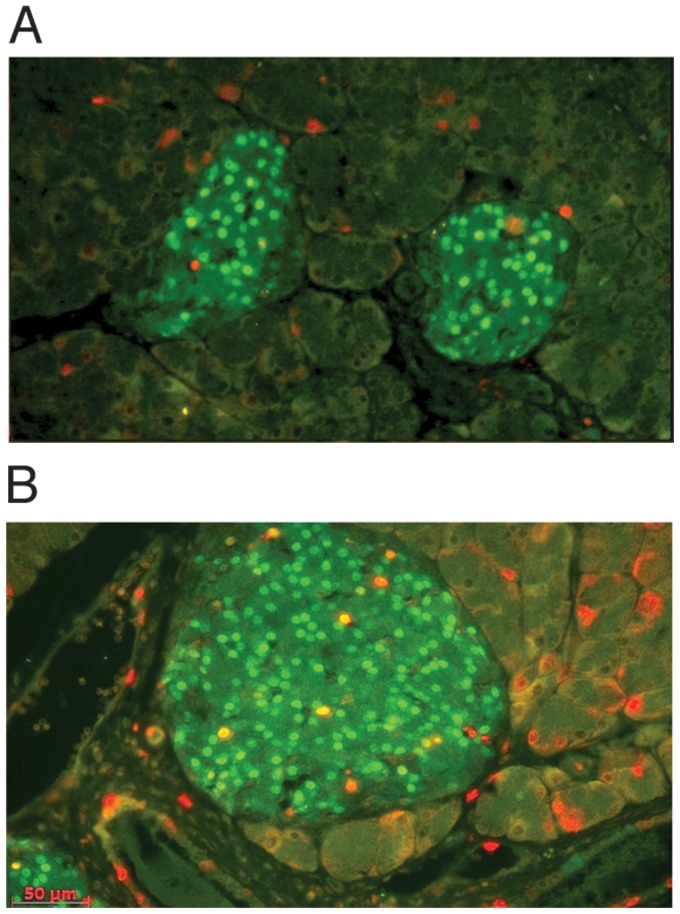
Cellular regeneration during recovery from RRV-induced pancreas involution. Pancreas sections from C57/B6 mice 7 days after inoculation with A) mock virus or B) RRV were immunostained for PCNA (red) and PDX-1 (green). PCNA-positive cells were increased following RRV, including scattered islet cells co-stained (yellow) for both PCNA and PDX-1. Magnification A: x10; B x20.

To obtain evidence that pancreatic cells had undergone regeneration following RRV-induced apoptosis and involution we stained the pancreas for expression of proliferating cell nuclear antigen (PCNA) and pancreas-duodenal homeobox 1 protein (Pdx1). Pdx1 is essential for pancreas development but is also required for the maintenance and regeneration of beta cells in the adult mouse pancreas [21]. By day 7 after inoculation with RRV, only an occasional TUNEL-positive cell was observed in the acinar pancreas but the number of PCNA-positive cells in both acinar tissue and islets had increased by over 2-fold compared to mock virus control mice (Figure 6), consistent with regeneration. As with TUNEL staining, there was no obvious difference in the distribution density of PCNA per area of acinar tissue or islets. As expected, islet cells expressed Pdx1, but following RRV some co-expressed PCNA indicating that they had undergone proliferation.

### RRV localizes to the pancreas in the second phase of hyperglycemia

One day after inoculation of C57/B6 mice, RRV measured by titration was detected mainly in the jejunum, but also in the serum and to a lesser extent in mediastinal lymph nodes and occasionally in the liver (Table 1). By day 3, the virus titer had increased in the jejunum and liver, was unchanged in mediastinal lymph nodes and had decreased in serum. By day 9, virus titer was highest in pancreas and spleen, and had decreased in jejunum. By day 14, virus was detected only at low titer in the pancreas and jejunum, and thereafter was undetectable. The titer of virus in the jejunum, mediastinal lymph nodes or liver did not correlate with blood glucose concentration (data not shown).

**Table 1:** Titers of rhesus rotavirus in tissues after inoculation of C57/Bl6 mice.

	Day 1	Day 3	Day 9	Day 14
Serum	10^3^ *	10^2^	0	0
Jejunum	10^4^	10^6^	10^3^	<10
Mediastinal lymph node	10^2^	10^2^	0	0
Pancreas	0	0	10^4^	10
Spleen	0	0	10^4^	0
Liver	<10	10^5^	0	0

* Titers are median values derived from fluorescence focus forming units on MA104 cells (3–6 mice/group).

**Figure 7:**
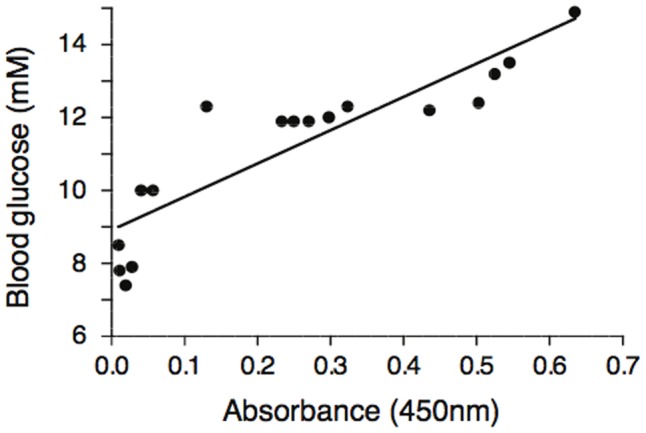
Correlation of blood glucose concentration with RV antigen in the pancreas. RV was quantified by ELISA 9 days after inoculation of C57/B6 mice with RRV.

By day 9 after inoculation with RRV, when pancreas mass and islet size had recovered (although many islets were not completely regular in outline), RRV was localized to the pancreas and the concentration of RRV antigen in the pancreas measured by ELISA strongly correlated with blood glucose concentration (r = 0.89, P<0.001) (Figure 7). Although the blood glucose concentration in RRV-inoculated mice had increased again and was greater than in mock virus control mice (12.8±1.63 vs 10.5±1.23, P = 0.02) plasma insulin concentration did not differ between the groups (0.56±0.29 vs 0.56±0.20 µg/l). H&E staining of the pancreas on days 9–14 revealed no lymphoid-inflammatory cell infiltration.

## Discussion

RV has been implicated as a trigger of type 1 diabetes [5], [7], [8]), acute pancreatitis [22]–[24] and acute fulminant diabetes [25]. Several strains of RV, including RRV, have been shown to infect mouse, fetal pig and monkey pancreatic islets in tissue culture [13] but direct experimental evidence for pancreas pathology after RV infection *in vivo* has not been reported previously. To our knowledge, the only other virus shown to cause pancreatic pathology after infection of mice is Coxsackie virus B4 [3], but the mechanism of the virus effect was not studied at the time. We observed a dramatic change in the integrity of the pancreas following inoculation of weanling C57/Bl6 mice with RRV, a type A, serotype G3 RV with strong sequence similarity to RV strains that infect humans. RRV triggered marked apoptosis throughout the pancreas associated with involution of the organ. Islets were strikingly smaller and irregular and insulin expression and secretion were reduced, accompanied by mild hyperglycemia. Evidence for direct infection of the pancreas at this time was lacking. Rather, the requirement for double stranded RNA in the form of inactivated RRV, not necessarily live virus, the similar effect of poly I∶C, and the absence of an effect of RRV in *TLR3* −/− mice, demonstrated that this immediate effect of RRV infection was mediated by the interaction of RRV double stranded RNA with TLR3. Double-stranded RNA was shown to induce apoptosis in cultured islets of C57/B6 mice via TLR3 and IFN regulatory factor 3 pathways [12] and repeated injections of poly I∶C have been shown to induce pancreatitis in autoimmune disease-prone MRL-Mp mice [26]. Our finding that the pro-apoptotic effect of RRV on the pancreas was not limited to islets is consistent with the widespread expression of TLR3 in the pancreas [27]. In addition to a direct TLR3-mediated effect in the pancreas, IFNs generated via TLR3 from extra-pancreatic sites could also contribute to pancreatic cell death, given that the death of gut epithelial cells after RRV infection was blocked by antibody to IFN-β [28]. The rapid recovery of the pancreas after the initial RRV-induced insult, associated with cellular proliferation, represents a potential model for studying pancreas regeneration. Following recovery, a second phase of mild hyperglycemia coincided for the first time with the presence of virus in the pancreas, but without an inflammatory cell infiltrate. At this time, the plasma insulin concentration in mice inoculated with RRV was similar to that in control mice, although it can be argued that it was inappropriately low relative to the mild hyperglycemia in these mice. Further studies are required to determine if RV in the pancreas impairs insulin secretion, how virus is cleared from the pancreas and the influence of genotype and immune status on the response to virus.

Hyperglycemia was mild and transient following inoculation with RRV, possibly reflecting rapid recovery of the pancreas after the acute insult. Repeated infection with RV, as occurs in children, might result in a more marked and sustained effect. Moreover, the response to RV infection, including any propensity to develop islet autoimmunity, is likely to be determined by the immunogenetic background. In NOD mice, which are genetically prone to immune dysregulation and autoimmune diabetes, RV infection in the neonatal period retarded [29], [30] but at [29] or after [14] weaning accelerated the development of diabetes. However, in these and a further study in NOD mice that described T helper-1 cytokine expression in pancreatic lymph nodes after RRV infection [31], the condition of the pancreas was not reported. Acting as an innate immune trigger, RV double stranded RNA could potentially set the stage for adaptive immunity to beta cells in genetically susceptible hosts. In his seminal description of the pathologic anatomy of the pancreas, Gepts [32] noted that the pancreas in type 1 diabetes was smaller than normal. A contemporary report [33] has now revealed that a decrease in pancreas size precedes clinical presentation of type 1 diabetes in humans. Extrapolated to humans, our observations would provide a rationale to monitor RV infection in children in relation to acute pancreatitis and/or development of islet autoimmunity, and to assess the impact of the recently introduced RV vaccine on the incidence of these disorders.
